# Publisher Correction: Analysis and optimization of quantum adaptive measurement protocols with the framework of preparation games

**DOI:** 10.1038/s41467-022-27994-6

**Published:** 2022-01-14

**Authors:** M. Weilenmann, E. A. Aguilar, M. Navascués

**Affiliations:** 1 Institute for Quantum Optics and Quantum Information (IQOQI) Vienna Austrian Academy of Sciences, Vienna, Austria; 2 AIT Austrian Institute of Technology GmbH, Vienna, Austria

**Keywords:** Quantum information, Quantum mechanics

Correction to: *Nature Communications* 10.1038/s41467-021-24658-9, published online 27 July 2021.

In the original PDF of this article, figures 4 and 5 were inadvertently omitted. The original article PDF has been corrected. The HTML version was unaffected.

